# Coincidence of primary adrenocortical carcinoma and melanoma: three CASE reports

**DOI:** 10.1186/s12902-022-01253-7

**Published:** 2023-01-06

**Authors:** Ye Lynn Ko, Vaishnavi Kumar, Juliane Lippert, Salvador Diaz-Cano, Kassiani Skordilis, Otilia Kimpel, Stefan Kircher, Miriam Asia, Yasir S. Elhassan, Barbara Altieri, Cristina L. Ronchi

**Affiliations:** 1 Department of Endocrinology, Queen Elizabeth Hospital, University Hospitals Birmingham NHS Foundation Trust, Birmingham, UK; 2 Division of Endocrinology and Diabetes, Department of Internal Medicine I, University Hospital, University of Würzburg, Würzburg, Germany; 3 Department of Histopathology, University Hospitals Birmingham NHS Foundation Trust, Birmingham, UK; 4 Institute for Pathology, University of Würzburg, Würzburg, Germany; 5 Institute of Metabolism and System Research, College of Medical and Dental Sciences, University of Birmingham, Edgbaston, B15 2TT UK; 6 Centre for Endocrinology, Diabetes, and Metabolism (CEDAM), Birmingham Health Partners, Birmingham, UK

**Keywords:** Adrenal gland, Adrenocortical cancer, Melanoma, Differential diagnosis

## Abstract

**Background:**

Adrenocortical carcinoma (ACC) is a rare endocrine malignancy with a heterogeneous prognosis, while adrenal metastasis from other primary cancers, including melanoma, may occur more frequently. ACC may rarely occur as part of familial cancer syndromes, but even in sporadic cases, a significant proportion of patients had other malignancies before or after diagnosis of ACC. Herein we present three cases where sporadic ACC was identified in patients with coexistent or previous history of melanoma.

**Case description:**

Patient 1 - A 37-yr-old man with a superficial spreading *BRAF*-positive melanoma was found to harbour a progressively growing left adrenal mass. Initially, he was suspected of having adrenal metastasis, but the histology after adrenalectomy confirmed ACC. Patient 2 - A 68-year-old man with a history of recurrent *BRAF*-positive melanoma was diagnosed with disseminated metastatic melanoma recurrence, including a rapidly enlarging left adrenal mass. Consequently, he underwent left adrenalectomy, and histology again confirmed ACC. Patient 3 – A 50-yr-old man was referred with histological diagnosis of metastatic ACC. He had a background history of pT1 melanoma.

We undertook targeted sequencing of ACC tissue samples in all cases. Somatic variants were observed in the known driver genes *CTNNB1 (*Patient 1), *APC* and *KMT2D* (Patient 2), and *APC* and *TP53* (Patient 3). Germline *TP53* variants (Li-Fraumeni syndrome) were excluded in all cases.

Retrospective review of our patient cohort in the last 21 years revealed a frequency of 0.5% of histologically diagnosed melanoma metastasis among patients referred for adrenal masses. On the other hand, 1.6% of patients with histologically confirmed ACC had a previous history of melanoma.

**Conclusion:**

Sporadic ACC can occur in the background of melanoma, even if adrenal metastasis might appear to be the most likely diagnosis. Coexistent primary adrenal malignancy should be considered and investigated for in all patients with a history of melanoma with suspicious adrenal lesions.

**Supplementary Information:**

The online version contains supplementary material available at 10.1186/s12902-022-01253-7.

## Background

Adrenocortical carcinoma (ACC) is a rare and aggressive endocrine malignancy with an incidence of 0.7–2.0 cases/million/year. It commonly arises sporadically, but in rare cases, may also be a part of familial syndromes [1, 2]. ACC has been traditionally linked to Li-Fraumeni syndrome (LFS), an autosomal dominant disorder, caused by germline variants in the suppressor gene *TP53,* where co-tumours, such as leukemia, sarcomas, brain tumours, and breast cancers, are often seen [3, 4]. Overall, ACC develops in 6–13% of individuals with LFS [5]. ACC can occur in the context of other hereditary genetic syndromes such as Beckwith-Wiedemann syndrome, Von Hippel-Lindau disease, multiple endocrine neoplasias (types 1 and 2), Carney complex, Lynch syndrome, or hereditary non-polyposis colorectal cancer and familial adenomatous polyposis [6–9]. Even outside recognised familial cancer syndromes, sporadic ACC can arise synchronously with or in the background of other malignant tumours, such as testicular and ovarian tumours, rectal cancer, stomach gastrointestinal stromal tumour, osteosarcoma, rhabdomyosarcoma, myoepithelioma, and neuroblastoma [10–13]. However, to our knowledge, coexistence or direct association between ACC and melanoma has never been reported.

Melanoma is the most aggressive type of skin cancer, arising from the pigment-producing cells melanocytes. The adrenal gland is one of the most common sites of metastasis after the lung, liver, and bone [14]. Most cases of melanoma are sporadic while the most frequent somatic mutation occurs in the 600th codon of the gene *BRAF* (50% of cases) [15]. Oncogenic RAS genes (*NRAS, HRAS*, and *KRAS*) are also recurrently mutated (30% of The Cancer Genome Atlas cases) [16]. Loss-of-function mutations can also affect tumor suppressor genes, such as *NF1, TP53*, and *CDKN2A*, mutually exclusive to *BRAF*.

The genetic background of ACC is mainly heterogeneous. The most common alterations involve the activation of the Wnt/β-catenin pathway (i.e., variants in *ZNRF3, CTNNB1,* and *APC*) or the p53 apoptosis/Rb1 cell cycle (i.e., variants in *TP53* and *CDKN2A)* [17–20]*.* Additionally, loss of heterozygosity at the 11p15 locus, leading to loss of maternal imprinting and increased expression of IGF2, is observed in up to 85% of adult ACC [19, 21, 22]. More rarely, alterations in chromatin remodelling/maintenance and PKA/cAMP signalling have also been reported [23, 24].

Herein, we present three cases where ACC was identified in patients with a history of melanoma, from two European Centres. For all cases, targeted sequencing of ACC tissue was undertaken. We also retrospectively studied the case records of patients assessed at a large tertiary adrenal tumour service in the UK in a 21 year period (2000–2021) to further evaluate the coexistence of ACC and melanoma. This presented an opportunity to reflect on the differential diagnosis of malignant adrenal lesions, particularly in the context of cancer that can metastasise to the adrenal gland.

## Case presentation

Demographic, clinical, histopathological, and hormonal data of the presented cases are reported in Table 1. Representative haematoxylin-eosin staining samples of the ACC are shown in Fig. 2.

**Table 1:** Demographic, clinical data and hormonal tests at the time of diagnosis of adrenocortical carcinoma

Characteristic	Case 1	Case 2	Case 3
Sex/Age (yrs)	M/37	M/68	M/50
**Biochemistry at diagnosis**			
Sodium - mmol/L (nr)	142 (133–146)	137 (133–146)	138 (133–146)
Potassium - mmol/L (nr)	4.2 (3.5–5.3)	4.6 (3.5–5.3)	5.0 (3.5–5.3)
Creatinine - μmol/L (nr)	91 (64–104)	85 (64–104)	76 (0–117)
eGFR - mL/min (nr)	82 (> 60)	78 (> 60)	107 (> 60)
**Endocrine workup at diagnosis**			
Aldosterone - pmol/L (nr)	286 (< 750)	NA	NA
Renin - mU/L (nr)	35.1 (4.2–59.7 supine)	NA	NA
Cortisol post 1 mg dexamethasone - mmol/L (nr)	< 28 (< 50)	NA	NA
ACTH - ng/L (nr)	27.5 (0–46)	NA	NA
Androstenedione - nmol/L (nr)	5.8 (1.1–5.6)	1.6 (1.1–5.6)	NA
Testosterone - nmol/L (nr)	23.1 (7.0–27.0)	18.9 (7.0–27.0)	NA
DHEAS - μmol/L (nr)	10.88 (3.80–13.10)	NA	NA
17OHP - nmol/L (nr)	2.5 (1.2–3.7)	3.1 (1.2–3.7)	NA
Plasma or urinary metanephrines	Normal	NA	NA
**Family history**	Mother breast cancer Brother lung cancer	Negative	One brother melanoma One brother testis cancer
**Histology of melanoma**			
Initial staging	pT3b	metastatic	pT1a
BRAF status	c.1799 T > A, p.V600E	c.1799 T > A, p.V600E	Unknown
**Histology of ACC**			
Initial staging	pT2 dedifferentiated	pT3 L1 V1 N0 blastematous	pT3 M1
ENSAT tumour stage	2	3	4
Weiss score	4	unknown	9
Ki67 index	11%	80%	50%

*eGFR* estimated glomerular filtration rate; *ACTH* adrenocorticotrophic hormone; *DHEAS* dehydroepiandrosterone sulphate; 17*OHP* 17 hydroxyprogesterone; *NA* not available, *nr* normal range

**Fig. 1:**
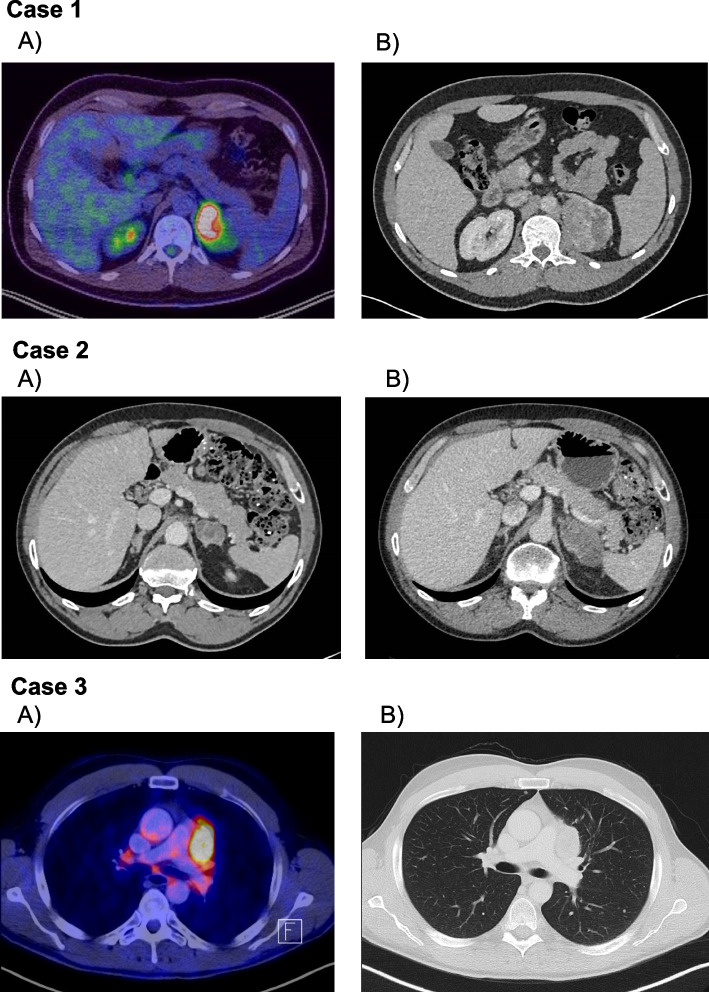
Radiological imaging of large adrenocortical lesions before surgery or at the time of disease recurrence. Case 1 (at time of diagnosis). Panel **a**) FDG-PET CT scan December 2019: The left adrenal mass is concordant with the CT scan from November. The lesion shows abnormal FDG uptake with maximum S U V is 16.2. The lesion remains highly suspicious of soft tissue metastasis from melanoma. Panel **b** Abdomen Pelvis CT scan April 2020: The left adrenal mass measures up to 73 mm maximum axial dimension. It is closely related to the medial aspect of the spleen where direct invasion cannot be excluded, to the left crus of the left kidney but there are thought to be thin planes of separation from these structures. No evidence of other metastatic disease.of the diaphragm and to the upper pole. Case 2 (at time of diagnosis). Panel **a** and Panel **b**) Thorax Abdomen Pelvis CT scan October 2020 – Panel **b** (compared to July 2020 – Panel **a**): Further enlargement of the isolated multicystic left adrenal metastasis, now 62.0 mm × 32.5 mm (compared to 33 × 26 mm in 3 months’ interval). The metastasis now abuts the stomach and spleen as well as the pancreas. Multi-planar reconstruction suggests the stomach rugae are lying against the metastasis rather than being invaded by it. No evidence of other metastatic disease. C) Case 3 (at time of disease recurrence). Panel **a**) FDG-PET CT scan and **b**) Abdomen Pelvis CT scan March 2015 at time of tumor progression. Evidence of lung metastases and new lymph nodes metastasis in the mediastinum

Case 1: A 37-year-old man seen by Dermatology for a superficial spreading, *BRAF*-positive malignant melanoma on his right flank diagnosed in May 2019. His mother developed breast cancer at the age of 30 years and his brother developed lung cancer aged 48. He underwent an excisional biopsy of the skin lesion, and the histology was consistent with tumour stage pT3b, sentinel lymph node-negative, and positive somatic *BRAF* pathogenic variant (c.1799 T > A, p.Val600Glu). He underwent a Thorax-Abdomen-Pelvis (TAP) CT scan in November 2019, which detected a 6 cm left adrenal mass and multiple small lung nodules. A subsequent Positron Emission Tomography CT (PET-CT) showed avid FDG uptake in the adrenal lesion, but not in the lung nodules. Surveillance TAP CT scans showed a significant increase in the size of the adrenal lesion to 7.2 cm over 6 months, which was therefore considered suspicious for adrenal metastasis (Fig. 1 – Case 1). The tiny lung nodules remained stable. He was referred to our Adrenal Multi-Disciplinary Team (MDT) meeting for further investigations. To differentiate between adrenal metastasis and primary ACC, we performed a complete adrenal workup, including overnight dexamethasone suppression test, adrenal androgens, and plasma metanephrines and normetanephrines, which did not show any adrenal hormonal excess (Table 1).

**Fig. 2:**
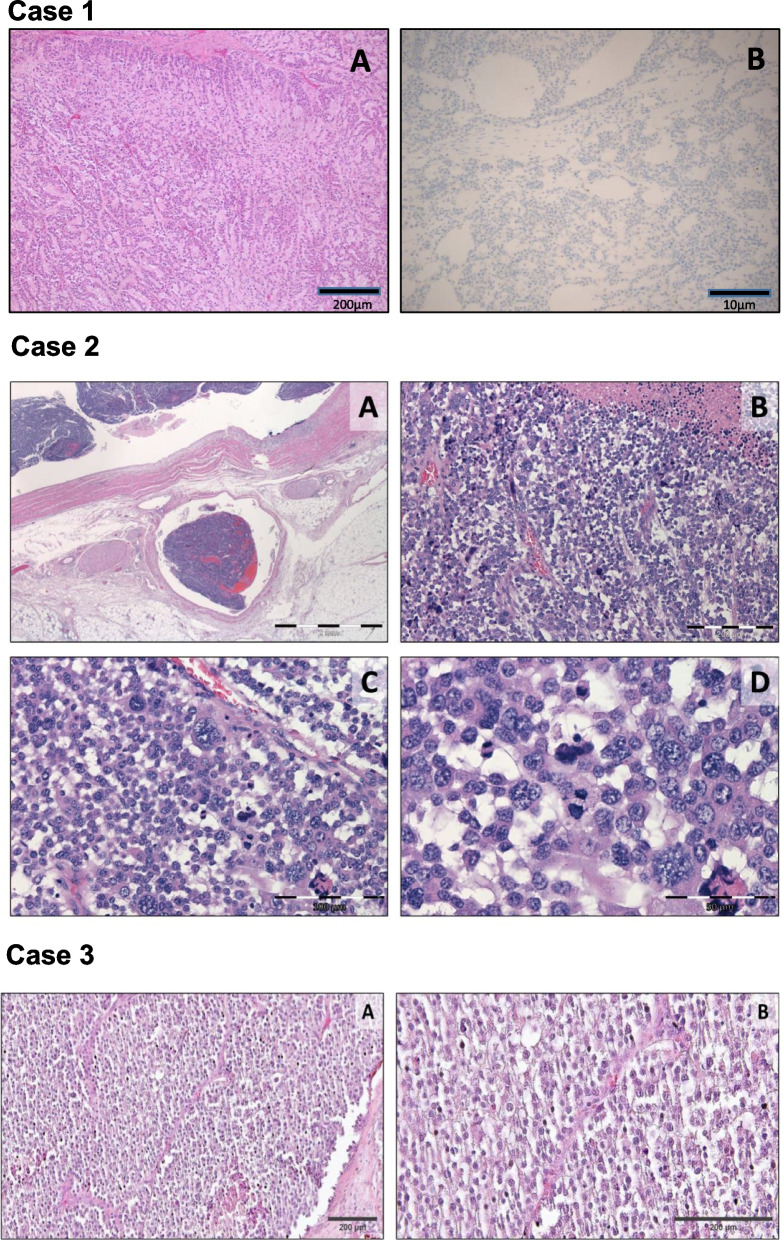
Representative histological pictures – Immunohistochemistry patterns of the three adrenocortical carcinomas. Case 1. Adrenocortical carcinoma composed of trabeculae and cords of medium cells with eosinophilic cytoplasm dispersed within myxoid stroma (panel A, H&E, 5x). The tumour was negative to Melan A (panel B, Melan A, 10x). Slides were imaged with the Leica Aperio Versa brightfield scanning microscope (Leica Biosystems, Wetzlar, Germany) and pictures were obtained using the Aperio ImageScope software (Leica Biosystems). Scale bar = 200 μm and 10 μm, respectively. Case 2. Adrenocortical carcinoma with blastematous histological features. The neoplasm revealed extra-adrenal extension, venous vascular invasion (panel A, H&E 12.5x), high nuclear/cytoplasmic ratio with confluent necrosis (panel B, H&E, 100x), frequent mitotic figures (panel C, H&E 200x), and numerous atypical mitoses (panel D, H&E 400x). The images were acquired using the ZEN core software (version 3.1). The minimal resolution was 1.375 μm for 4x magnification, 0.61 for 10x, 0.37 for 20x, and 0.29 for 40x. Scale bar = 2 mm, 200 μm, 100 μm and 50 μm, respectively. Case 3. Adrenocortical carcinoma with infiltration of the liver. The microscopic report described a diffuse tumour architecture with necrosis, tumour cells with a high nuclear grade, mitotic count of > 20 mitoses/50 HPF, atypical mitotic figure, sinusoidal, vascular, and capsular invasion. H&E slides were imaged with the Leica Aperio Versa brightfield scanning microscope (Leica Biosystems, Wetzlar, Germany) and picture were obtained using the Aperio ImageScope software (Leica Biosystems) with 10x (panel A) and 20x (panel B) enlargement. Scale bar = 200 μm. All the images have been acquired without filters, additional processing, or adjustment for size or color

The patient underwent left adrenalectomy, for a presumed oligometastasis from melanoma, in May 2020. Histology revealed a microscopic appearance and molecular and immunohistochemical profiles completely different from a malignant melanoma. Instead, light microscopy appearances and electron microscopy findings were compatible with a primary adrenocortical origin tumour. The tumour showed patchy synaptophysin and diffuse NSE expression but was negative for all applied melanoma markers (melan-A, Fig. 2 – Case 1, S-100, HMB-45, SOX10) and other adrenocortical markers (inhibin, calretinin). Slides were imaged with the Leica Aperio Versa brightfield scanning microscope (Leica Biosystems, Wetzlar, Germany) and pictures were obtained using the Aperio ImageScope software (Leica Biosystems) (Fig. 2a). The tumour did not express SMA, desmin, CD31, CD34, CD17, or chromogranin. There was no loss of SNF5. It was concluded that this was a de-differentiated ACC (pT2) with Weiss score 4, modified Weiss score 5, Ki-67 index 10–11%, completely resected (R0).

Case 2: A 68-year-old man with a history of recurrent melanoma and three previous resections in 2001 (right lower back) and April 2013 (right upper back and right upper abdomen), presented in March 2019 with suspected disease recurrence in the right axilla and subcutaneous nodules in the thoracic region. Genetic investigations were not previously performed to investigate the presence of somatic *BRAF* variants. There was no family history of cancer. Biopsy confirmed metastatic melanoma positive for a pathogenic variant in codon 600 of the *BRAF* gene (c.1799 T > A, p.Val600Glu). A staging TAP CT scan revealed disseminated metastatic disease with multiple subcutaneous, mediastinal and peritoneal nodules, indeterminate liver deposits and bilateral adrenal lesions.

The patient was commenced on a combination of Nivolumab and Ipsilumumab in August 2019. His metastatic melanoma showed an excellent response to immunotherapy with disappearance of the nodal and right adrenal disease manifestation, apart from the left adrenal mass which increased in size from 19 mm to 62 mm on serial surveillance imaging (Fig. 1 – Case 2). Following this rapid increase in size of the presumed left adrenal metastasis, which was inconsistent with the response to immunotherapy in the rest of the body, a decision was made to proceed with left adrenalectomy (December 2020).

The histology of the resected adrenal mass did not show any features consistent with melanoma but rather showed an ACC (pT3 L1 V1 N0) with high histological grade and blastematous characteristics (Fig. 2- Case 2). The tumour cells revealed focal cytoplasmic expression of calretinin and synaptophysin, and they were negative for S100, HMB45, melan A, and chromogranin. The Ki-67 index was 80% with incomplete resection status (R1). The shown microscopy images were acquired using the ZEN core software (version 3.1). The minimal resolution was 1.375 μm for 4x magnification, 0.61 for 10x, 0.37 for 20x, and 0.29 for 40x (Fig. 2b).

Case 3: A 50-year-old man came to our attention 1 month after the radical surgical resection of a right ACC associated with single liver metastasis (ENSAT stage IV). He presented a few months earlier with an incidental retroperitoneal lesion, infiltrating the liver, and dyspnoea. Urinary normetanephrine and metanephrine were within the normal range but no other hormonal diagnostic workup was performed before adrenalectomy. Histology reported described a 225 mm necrotic adrenal lesion with infiltration of the liver segment VIII and 25 mm single liver metastasis within segment VIII/IV (Fig. 2 - Case 3). Immunohistochemistry for SF1, inhibin, and melan A was positive and was therefore consistent with ACC (pT3, M1), Weiss score of 9 and Ki-67 index of 50%. The shown H&E slides were imaged with the Leica Aperio Versa brightfield scanning microscope (Leica Biosystems, Wetzlar, Germany) and picture were obtained using the Aperio ImageScope software (Leica Biosystems) (Fig. 2c).

The patient reported that he underwent a resection of a 0.72 mm melanoma of the left shoulder (pT1a, N0, M0) 11 years earlier. His brother also suffered from melanoma and another brother had a history of testicular cancer.

### Treatment and follow-up

Case 1: The patient was commenced on adjuvant mitotane therapy in June 2020 in line with the local and international guidelines [25]. His surveillance imaging up to December 2021 did not show any local recurrence or distant metastasis. The known small indeterminate lung nodules have remained stable over time. The local Dermatology team is simultaneously following him to monitor the melanoma.

Case 2: The patient was commenced on adjuvant mitotane therapy in January 2021 in line with the local and international guidelines [25]. However, mitotane had to be permanently discontinued due to significant hepatotoxicity. Oncologists continue to closely monitor his melanoma. Surveillance TAP CT scans until December 2021 showed no recurrence of his ACC and stable appearances of the right lower lobe subpleural nodule.

Case 3: Because of the high risk of recurrence, the patient was treated with 4 cycles of adjuvant carboplatin and etoposide [26]. After tumour progression with two lung metastases, etoposide-doxorubicin-platinum associated with mitotane were started [27], but unfortunately without any objective response. He was subsequently treated with 8 cycles of gemcitabine plus capecitabine [28] and radiotherapy to one metastasis in the mediastinum. In March 2015, FDG-PET/TC scan showed progression of the lung metastases and the development of a new lymph node metastasis in the mediastinum (Fig. 1 – Case 3). Mitotane therapy was discontinued, and he was commenced on streptozotocin chemotherapy. His disease remained stable and streptozotocin was continued for 21 cycles but then he developed new liver metastasis in segment II. In April 2016, the patient underwent resection of the liver metastasis and resection of thoracic metastases 1 month later. He remained tumour-free until December 2021.

### Genetic analysis

#### Genetic counselling

Due to two different types of malignancies, genetic counselling was recommended for patient 1 (additional family history for other cancer types) and 2. Genetic testing for *TP53* gene alterations (screening for Li-Fraumeni Syndrome) was performed and came back negative in both cases. Genetic counselling was not undertaken for Case 3.

#### Targeted next-generation sequencing (NGS)

Targeted DNA sequencing was undertaken on the three tumour samples as part of a previous study (Case 3, [20] or an ongoing collaborative project (Case 1 and 2, data unpublished). FFPE ACC tumour material and EDTA blood samples were collected to investigate somatic variants by targeted next-generation sequencing (NGS). Tumour-DNA and leukocyte were isolated using commercially available kits (GeneRead DNA FFPE Kit, Qiagen, Hilden, Germany, and NucleoSpin Blood L Kit (Macherey-Nagel, Bethlehem, PA), respectively. Samples were either enriched with a Cell3 Target Custom Panel (Nonacus, Birmingham, United Kingdom) – including 33 genes known to be associated with ACC (gene list provided in Suppl Table 1) – (Case 1 and 2) or with the Human Comprehensive Cancer Panel (Qiagen) – including a total of 160 cancer-related genes (Case 3) [20]. Sequencing was performed on a NextSeq500 with Mid Output Reagent Kit V2 and 150 cycles paired-end read sequencing or on a MiSeq with 300 cycles paired-end sequencing (Illumina Inc., San Diego, CA, USA), respectively. Raw data were aligned and analysed with GensearchNGS (Phenosystems S.A., Belgium). For detection of single nucleotide variants (SNV) and small insertions and deletions (small Indels) the called variants were filtered as followed: exon distance < 21; variant allele frequency (VAF) > 0.1; Minor Allele Frequency (MAF) < 0.02; variant balance > 0; Type = worse than synonymous [20]. By comparing data from tumour and blood samples, variants were classified as somatic (detectable only in tumour sample) or germline (detectable in tumour and blood sample).

## Results

A detailed summary of all the observed variants is shown in Table 2.

**Table 2:** Results of targeted sequencing analysis in the three patients with adrenocortical carcinoma and a previous history of melanoma

	Type of gene panel	Variant (gene name and position)	Amino acid substitution	classification
**Case 1**	33 genes^a^	**Somatic:**		
		*CTNNB1:* NM_001904.3: c.133C > T	p. Ser45Pro	**likely oncogenic**
		**Germline: -**		–
**Case 2**	33 genes^a^	**Somatic:**		
		*APC*: NM_000038.5, c. 14C > T	p.Ser5Leu	**likely oncogenic**
		*KMT2D*: NM_003482.3, c.12935C > T	p.Ser4312Phe	**likely oncogenic**
		**Germline:**		
		*MLH1:* NM_000249.3: c.977 T > C	p.Val326Ala	**uncertain**
		*ATM:* NM_000051.3: c.2119 T > C	p.Ser707Pro	likely benign
**Case 3**	160 genes^b^	**Somatic:**		
		*APC*: NM_001127511, c.G4558T	p.E1520X	**likely oncogenic**
		*TP53*: NM_000546.5, c.365_366del	p.Val122Aspfs^a^26	**likely oncogenic**
		**Germline:**		
		*AKT2:* NM_001626.5:c.177A > G	p.Glu59=	
		*ATM:* NM_000051.3:c.7316 T > C	p.Val2439Ala	likely benign
		*CBLB:* NM_170662.4:c.2581G > T	p.Asp861Tyr	**uncertain**
		*CBLB:* NM_170662.4:c.1865G > C	p.Ser622Thr	**uncertain**
		*FANCD2* NM_001018115.2:c.78A > C	p.Gln26His	likely benign
		*FANCD2:*	p.Pro714Leu	likely benign
		NM_001018115.2:c.2141C > T	p.Asp1327Gly	benign
		*NOTCH2:* NM_024408.3:c.3980A > G	p.Lys541Glu	benign
		*PMS2:* NM_000535.6:c.1621A > G		benign

Somatic variants detected in formalin-fixed paraffin-embedded tissue material from adrenocortical carcinoma

^a^ Customised gene panel including 33 ACC-specific genes (see Suppl Table 1)

^c^ Commercially available gene panel including 160 cancer-related genes

Case 1: a somatic variant was found in a known disease-causing hot-spot region of gene *CTNNB1* (NM_001904.3, c.133C > T, p. Ser45Pro), leading to constitutively activated ß-Catenin. No germline variants in any of the 33 genes analysed by targeted NGS were detected.

Case 2: we observed two likely oncogenic somatic variants, one in *APC* (NM_000038.5, c. 14C > T, p.Ser5Leu) and one in *KMT2D* (NM_003482.3, c.12935C > T, p.Ser4312Phe). In addition, the targeted sequencing of DNA isolated from whole blood revealed germline variants in genes *MLH1* (NM_000249.3, c.977 T > C, p.Val326Ala) and *ATM* (NM_000051.3, c.2119 T > C, p.Ser707Pro), which were classified as a variant of uncertain significance and likely benign, respectively.

Case 3: two somatic variants were detected in *APC* (NM_001127511, c.4558G > T, p.E1520X) and *TP53* (NM_000546.5, c.365_366del, p.Val122Aspfs*26) both classified as likely oncogenic. Moreover, interestingly, in this case eight germline variants were observed in 6 different genes (out of 160 investigated), including *PMS2* (benign) and *ATM* (likely benign). Among these, only variants in gene CBLB was classified as of uncertain significance.

### Frequency of melanoma as second cancer in patients with ACC

We then retrospectively evaluated the cohort of patients with suspicious adrenal lesions presented at the Queen Elizabeth Hospital Birmingham between 2000 and 2021 to investigate the relevance of past medical history of melanoma. First, we looked at the frequency of history of melanoma, but without recognised familial cancer syndromes, in our cohort of patients with diagnosed ACC. We observed 2 out of 126 patients with a documented history of melanoma (1.6% of total). Second, we checked the frequency of diagnosed adrenal metastasis from primary melanoma among patients referred for indeterminate/suspicious adrenal masses. Altogether, only one out of 201 patients (0.5%) had a histological diagnosis of melanoma metastasis. This percentage was higher when considering only patients with a previous history of non-adrenal cancers (1 out of 48; 2%).

## Discussion and conclusion

We present three cases of ACC diagnosed in patients with a history of melanoma. In two cases, adrenalectomy was undertaken for a presumed oligometastasis from underlying malignant *BRAF*-mutated melanoma. However, histology of the adrenal lesions was consistent with primary adrenal cancer, and adjuvant treatment with mitotane was promptly started. In the third case, ACC occurred in a background history of low-aggressive melanoma.

The association of two primary malignancies could be approached from two perspectives. First, patients with malignant melanoma are known to increase the overall risk of second primary cancer [29, 30]. The type of malignancies reported differs depending on the subtype of melanoma. In general, patients with malignant melanomas had increased occurrence of thyroid cancer (cutaneous melanoma), renal cancer (non-acral, mucosal, and uveal melanomas), and lymphoma (non-acral and acral melanomas). The proposed biological rationale was the common oncogenic mutations of the mitogen-activated protein kinase pathway in cutaneous melanomas and thyroid cancers, the shared immunogenicity and BAP1 aberrations in kidney cancers, cutaneous and uveal melanomas, and the decreased immune surveillance for lymphomas and melanomas [31]. Second, the incidence of adrenal gland neoplasms as a second primary malignancy revealed a high incidence ratio in specific primary sites, including hypopharynx, other endocrine tissue (including the thymus), small intestine, liver, stomach, nodal non-Hodgkin lymphoma, kidney, and renal pelvis, and breast [32]. The available literature about adrenal gland neoplasms as a second primary cancer is limited; its incidence is higher than expected and does not mention the connection with malignant melanoma.

Choroid plexus tumours, sarcomas, early-onset breast cancers, brain cancers, and leukaemias, can typically co-exist with ACC within LFS, caused by known germline *TP53* mutations. Nevertheless, other cancers, such as lung adenocarcinoma, melanoma, gastrointestinal tumours (i.e., colon, pancreas), kidney, thyroid, and gonadal germ cells (i.e., ovarian, testicular, and prostate), may develop along with ACC in patients with less explored germline *TP53* mutations [4, 5, 33–35]. In addition, second malignancies have also been reported in ACC patients without hereditary cancer syndromes [36, 37]. In particular, Ayala-Ramirez et al. reported a frequency of 11.5% of patients with other cancers before or after their diagnosis of ACC. Breast and prostate cancers were the most common (both 18.4%), followed by skin cancer (10.5%), non-small cell lung cancer (10.5%), endometrial carcinoma (7.9%), papillary thyroid cancer (5.2%), renal cell carcinoma (5.2%), and melanoma (5.2%). However, no association or specific tumour pattern has been catalogued in previous studies, and there is no recognised link between ACC and melanoma.

On the other hand, in patients with a history of cancer, suspicious adrenal masses pose the crucial issue of differential diagnosis between adrenal metastasis and ACC [25]. In general, adrenal metastases are the most common malignant lesions affecting the adrenal gland and the second most common adrenocortical tumour after benign adenomas [38]. Almost any cancer can spread to the adrenal glands, but some tumours are more likely than others to metastasize to this region. The cancers that most frequently spread to the adrenal gland(s) are lung cancer, kidney cancer (renal cell carcinoma), and malignant melanoma, followed by breast, colon, and rectal cancer. Furthermore, post-mortem examination of patients with advanced-stage melanoma has found adrenal gland metastases in approximately 50% of cases, making the adrenal gland the fourth most common site of metastasis after lung, liver, and bone [14]. Therefore, adrenal metastasis would be the most likely diagnosis in a patient with a suspicious adrenal mass and previous history of melanoma. However, according to our case series and our casuistic review, where melanoma co-existed in 2.3% of ACC cases, and adrenal metastasis from melanoma was observed in 0.6% of patients with suspicious adrenal masses, the possibility of primary adrenal cancer should always be considered.

From a molecular perspective, two of our patients had somatic *BRAF* mutations detected at the malignant melanoma level. BRAF, serine/threonine kinase, plays a vital role in the RAS-MEK-ERK pathway that relays extracellular signals for cell proliferation and survival. Somatic variants of the *BRAF* gene are associated with 60% of malignant melanomas and moderate to high frequency in colorectal, ovarian, and papillary thyroid carcinomas [39, 40]. *BRAF* mutations have also been described in few cases of ACC. For instance, Kotoula et al. observed *BRAF* variants in two out of 35 ACC samples [41]. Our group also previously detected recurrent somatic alterations in genes not previously associated with ACC, including *BRCA1, BRCA2,* and *BRAF, i*n 9 out of 117 specimens analysed [20]. However, no *BRAF* variants were detected in ACC samples from the present small case series using targeted sequencing.

Of note, we identified a few somatic alterations in our three ACC samples. In all cases, we detected a somatic mutation in one Wnt/β-catenin related gene, with additional variants in *KMT2D* in Case 2 and *TP53* in Case 3. This finding agrees with previous pan-genomic studies that collectively revealed modification of the Wnt/β-catenin pathway in 41% of ACC cases [17, 19]. Specifically, in Case 1, a well-known, activating missense *CTNNB1* variant has been observed. This likely oncogenic mutation is described in different types of cancer [42–45] and about 30% of ACC [17, 19, 20]. In both other cases, somatic missense mutations in gene *APC,* coding for a tumor suppressor protein that acts as an antagonist of the Wnt/β-catenin signalling pathway, were detected. Furthermore, in Case 2, we observed an additional likely pathogenic, somatic missense mutation in gene *KMT2D*. KMT2D is a histone H3 lysine-4 methyltransferase required for FOXA1, PBX, and oestrogen receptor (ER) recruitment and activation. AKT binds and phosphorylates KMT2D, attenuating methyltransferase activity and ER function, whereas PI3K-alpha inhibition enhances KMT2D activity. This activity regulates remodelling and organization of chromatin and can potentially contribute to the blastematous appearance of the neoplasm. Somatic mutations in this genes have been previously reported in a small percentage of ACC [20]. Finally, in Case 3, we detected a deletion in the well-known tumour-suppressor gene *TP53.* Again, this is following previous studies, where somatic alterations in the p53 apoptosis/Rb1 cell-cycle pathway (i.e., *TP53, CDKN2A, RB1, CDK4,* and CCNE1) were altered in 45% of ACCs. Interestingly, this area overlaps with the neoplastic development and progression in metastatic melanoma.

In conclusion, a complete diagnostic workup should be carried out to exclude ACC in patients with rapidly enlarging adrenal masses, even in patients with current or previous history of melanoma. An experienced Adrenal MDT has a crucial role to ensure timely management and adequate monitoring.

According to our interesting findings on a small case series, we believe that a future large and systematic study on the occurrence of adrenal gland neoplasms as second primary cancers after melanoma (or vice versa) is needed.

## Supplementary Information

**Additional file 1: Table S1.** List of genes included in the customised panel for Next Generation Sequencing.

## Data Availability

The DNA sequencing dataset generated and/or analysed during the current study are available in the BioProject repository at the link http://www.ncbi.nlm.nih.gov/bioproject/910169 (BioProject ID PRJNA910169). The other datasets are available from the corresponding author on reasonable request.

## Abbreviations

17OHP: 17 Hydroxyprogesterone
ACC: Adrenal Carcinoma
ACTH: Adrenocorticotrophic Hormone
CEDAM: Centre for Endocrinology, Diabetes and Metabolism
CT: Computed Tomography
DHEAS: Dehydroepiandrosterone sulfate
DNA: Deoxyribonucleic Acid
eGFR: Estimated Glomerular Filtration Rate
ENSAT: European Network For The Study of Adrenal Tumours
FDG: Flurodeoxyglucose
FFPE: Formalin Fixed Paraffin Embedded
HBRC: Human Biomaterial Resource Centre
HNPCC: Hereditary Non-Polyposis Colorectal Cancer
HPF: High Power Filed
LFS: Li-Fraumeni Syndrome
MAF: Minor Allele Frequency
MDT: Multi-disciplinary Meeting
NGS: Next Generation Sequencing
NSE: Neurone Specific Enolase
PET: Positron Emission Tomography
SMA: Smooth Muscle Actin
TAP: Thorax Abdomen and Pelvis
TCGA: The Cancer Genome Atlas
VAF: Variant Allele Frequency
