# Supplementary material for: Coincidence of primary adrenocortical carcinoma and melanoma: three CASE reports
Source: BMC Endocr Disord. 2023 Jan 6;23:4. doi: 10.1186/s12902-022-01253-7 (PMC9817389; doi:10.1186/s12902-022-01253-7)
Supplement: Supplementary file 1 — Additional file 1: Table S1. List of genes included in the customised panel for Next Generation Sequencing. [file 12902_2022_1253_MOESM1_ESM.docx]

**Supplementary Table 1.** List of genes included in the customised panel for Next Generation Sequencing*.

| N | Gene name and pathway | |  |
| --- | --- | --- | --- |
| 1 | *APC* | Wnt/beta catenin pathway | Exons Only |
| 2 | *ATM* | Homologous DNA Repair | Exons Only |
| 3 | *ATRX* | Chromatin remodelling | Exons Only |
| 4 | *BRCA2* | Homologous DNA Repair | Exons Only |
| 5 | *CDK4* | Rb/p53 | Exons Only |
| 6 | *CDKN2A* | Rb/p53 | Exons Only |
| 7 | *CTNNB1* | Wnt/beta catenin pathway | Exons Only |
| 8 | *DAXX* | Chromatin remodelling | Exons Only |
| 9 | *EGFR* | Angiogenic Factors | Exons Only |
| 10 | *FGFR3* | Angiogenic Factors | Exons Only |
| 11 | *FGFR4* | Angiogenic Factors | Exons Only |
| 12 | *GNA11* | cAMP/PKA pathway | Exons Only |
| 13 | *GNAS* | cAMP/PKA pathway | Exons Only |
| 14 | *IL7R* | Others | Exons Only |
| 15 | *KDM6A* | Chromatin remodelling | Exons Only |
| 16 | *KDR* | Angiogenic Factors | Exons Only |
| 17 | *KMT2D* | Chromatin remodelling | Exons Only |
| 18 | *MDM2* | Rb/p53 | Exons Only |
| 19 | *MEN1* | Chromatin remodelling | Exons Only |
| 20 | *MLH1* | Mismatch DNA repair | Exons Only |
| 21 | *MSH2* | Mismatch DNA repair | Exons Only |
| 22 | *NF1* | Others | Exons Only |
| 23 | *NOTCH1* | Others | Exons Only |
| 24 | *PRKACA* | cAMP/PKA pathway | Exons Only |
| 25 | *PRKAR1A* | cAMP/PKA pathway | Exons Only |
| 26 | *PTCH1* | Others | Exons Only |
| 27 | *RB1* | Rb/p53 | Exons Only |
| 28 | *SETD2* | Others | Exons Only |
| 29 | *SMARCB1* | Chromatin remodelling | Exons Only |
| 30 | *TERT* | Chromatin remodelling | Exons Only |
| 31 | *TNFRSF14* | Others | Exons Only |
| 32 | *TP53* | Rb/p53 | Exons Only |
| 33 | *ZNRF3* | Wnt/beta catenin pathway | Exons Only |

*Cell3 Target Custom Panel (Nonacus, Birmingham, United Kingdom)
